# Feasibility and Accuracy of an RTMPose-Based Markerless Motion Capture System for Single-Player Tasks in 3x3 Basketball

**DOI:** 10.3390/s25134003

**Published:** 2025-06-27

**Authors:** Wen Zheng, Mingxin Zhang, Rui Dong, Mingjia Qiu, Wei Wang

**Affiliations:** 1 School of Athletic Performance, Shanghai University of Sport, Shanghai 200438, China; 2321852032@sus.edu.cn (W.Z.); qiu274788941@gmail.com (M.Q.); 2 Shanghai Key Lab of Human Performance, Shanghai University of Sport, Shanghai 200438, China; 3 Key Laboratory of Sport Skill and Tactic Diagnosis and Analysis of General Administration of Sport of China, Shanghai University of Sport, Shanghai 200438, China; 4 School of Physical Education and Sports Science, South China Normal University, Guangzhou 510000, China; 5 Shanghai Artificial Intelligence Laboratory, Shanghai 200240, China

**Keywords:** markerless motion capture, RTMPose, pose estimation, 3x3 basketball, player displacement, computer vision

## Abstract

Markerless motion capture (MMC) offers a non-invasive method for monitoring external load in sports where wearable devices are restricted; however, its validity in 3x3 basketball contexts remains unverified. The viability and measurement precision of a multi-camera RTMPose-based MMC system for single-player tasks in 3x3 basketball performance monitoring were evaluated in this study. Recorded on a standard half-court, eight cameras (60 fps) captured ten collegiate athletes executing basketball-specific activities including linear sprints, curved runs, T-tests, and vertical jumps. The 3D coordinates of hip and ankle keypoints were reconstructed from multiple synchronized camera views using Direct Linear Transformation (DLT), from which horizontal displacement and average speed were derived. These values were validated using tape-measure distance and time–motion analysis. The MMC system demonstrated high accuracy, with coefficients of variation (CVs) below 5%, mean bias under 3.5%, and standard error of estimate (SEE) below 3% across most tasks. Speed estimates revealed great consistency with time–motion analysis (ICC = 0.97–1.00; standardized change in mean [SCM] varied from trivial to small). The Bland–Altman graphs verified no proportional error and little bias. These results confirm the MMC system as a consistent, non-invasive method for gathering movement data in outdoor basketball environments. Future studies should assess the system’s performance during live competitive play with several athletes and occlusions and compare it to a laboratory-grade motion capture system.

## 1. Introduction

Three-on-three (3x3) basketball is a high-intensity, fast-paced team sport that debuted at the Tokyo 2020 Olympics after its approval in 2017 [1]. During the Olympic Games, the women’s 3x3 basketball games included an average of approximately 69 possessions, with each offensive–defensive transition averaging mere 8.69 s, as inferred from the official statistics of 34 matches published on the FIBA 3x3 website [2]. In such a high-intensity competition environment, achieving real-time and accurate monitoring of athletic load to support evidence-based coaching and tactical decision-making has become a significant concern.

Currently, common load-monitoring methods in basketball include optical-tracking systems, video analysis (e.g., time–motion analysis, TMA), wearable sensors, and physiological or psychological assessments [3,4,5,6,7]. However, 3x3 basketball is typically played on temporary outdoor courts where fixed camera setups are not feasible, and game regulations restrict the use of wearable devices by players, limiting the applicability of existing methods.

Advances in computer vision have fostered markerless motion capture (MMC) systems that recover three-dimensional body kinematics directly from multi-camera video without reflective markers or on-body sensors [8,9,10,11,12]. Common acquisition techniques in markerless motion capture studies are depth cameras, monocular cameras, and multi-camera configurations. While monocular cameras find it difficult to produce 3D reconstruction from 2D postures, depth cameras are constrained by hardware price and operating range [13,14,15]. Existing studies have shown that markerless motion capture systems based on algorithms such as OpenPose and RTMPose can achieve centimeter-level accuracy across various sports [16,17], and thus can be applied for skill evaluation and movement pattern analysis in basketball [18], rugby [19], swimming [20], and others.

In sports settings, the real-time multi-person pose estimation algorithm (RTMPose) has demonstrated excellent accuracy and computational efficiency [21]. RTMPose is used to extract the target’s 2D keypoints, and then the Direct Linear Transformation (DLT) is applied to convert these coordinates into 3D keypoints, after which displacement can be quantified by tracking the keypoints. A recent study introduced the first publicly available comprehensive dataset for 3x3 basketball, demonstrating the feasibility of applying RTMPose to pose estimation in this sport. However, the accuracy of displacement tracking remains challenging [22].

MMC presents a viable substitute for wearable technology in tracking athlete movements, but its viability in 3x3 basketball has not yet been confirmed in real game conditions. There are still unresolved issues regarding the accuracy of the system in recording displacement outside of controlled laboratory environments, the effect of changing lighting conditions on keypoint detection, and the impact of frequent occlusions resulting from several players and referees moving in a small area [23]. It is still unknown if these elements compromise the accuracy of MMC system performance metrics.

This study aims to develop and test a markerless motion capture system for single-player tasks in 3x3 basketball using the RTMPose algorithm and analyze how well it can accurately track athletes’ movement, speed, and jump height on the court. The study investigates the possible use of the system for tracking workload during sport-specific training by assessing its measurement accuracy in usual movement patterns of 3x3 basketball. This study aims to provide a methodological foundation and technical support for data collection and load evaluation in future 3x3 basketball training and competition.

## 2. Materials and Methods

### 2.1. Participants

Ten collegiate 3x3 basketball athletes (age 23.3 ± 3.3 years; height 183.2 ± 14.5 cm; body mass 78.3 ± 16.7 kg) participated in this study. All participants provided written informed consent before the experiment, confirmed no history of major injuries, and could complete basketball-related physical tasks. This study was conducted in accordance with the Declaration of Helsinki and approved by the Shanghai University of Sport.

### 2.2. Equipment and Venue

**Figure 1:**
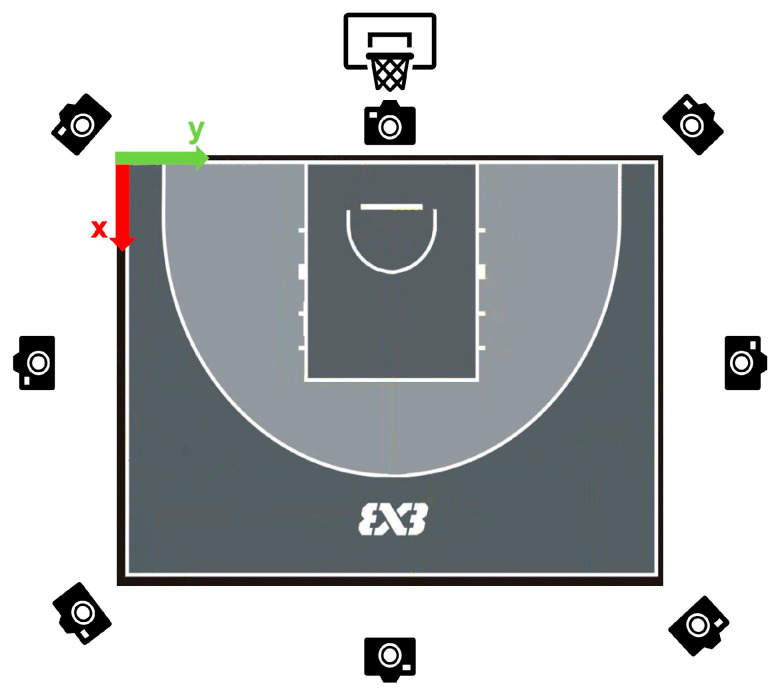
Placement and orientation of eight sports cameras surrounding the outdoor 3x3 basketball court used for markerless motion capture (Remarks: The origin of the world coordinate system was established at the left baseline corner when facing the basket, indicated by the X and Y horizontal axes in the upper-left corner, while the Z-axis is vertical, upwards from the ground).

Data collection took place on the outdoor 3x3 half-court (11 m × 15 m) of Shanghai University of Sport. Eight DJI Action 3 cameras (1920 × 1080 px, 60 fps, with a 16:9 aspect ratio) were mounted on two-meter tripods and evenly distributed around the court perimeter (Figure 1). The DJI Action 3 adopts a fixed-focus design and does not support auto-focus. To ensure consistency across all camera views, we standardized the recording parameters before data collection.

Cameras were synchronized with a remote-triggered audible clap. We stored the video data on MicroSD cards and then brought it into a central workstation for further processing. Two analysts (≥2  years of video analysis experience) employed MyVideoAnalyser v3.5.13 on MacBook Air M1 laptops to edit the footage, as detailed below.

Due to equipment constraints, tape measures and manual time–motion analysis were employed as pragmatic on-court references; higher-precision optical-marker validation will be addressed in future work.

### 2.3. Algorithm Workflow

**Figure 2:**
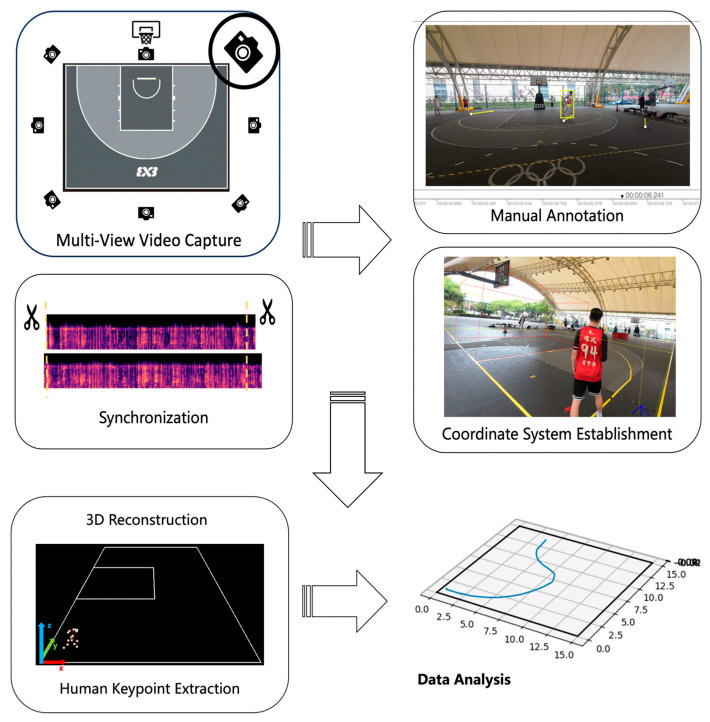
Algorithmic workflow of the RTMPose-based markerless motion capture system. The world coordinate system was established with the left baseline corner (facing the basket) as the origin.

The software algorithm in this study consisted of the following main steps (Figure 2).

We used Mel Frequency Cepstral Coefficients (MFCCs) to extract audio features to synchronize the video recordings, because it accurately captures the spectral envelope of sound signals and models the human auditory perception system. It is reliable and effective at detecting brief, sharp acoustic events for synchronization purposes, even in noisy settings [24]. The world coordinate system was established using the left-side baseline corner of the basketball court as the origin, referencing court markings and standard court dimensions. Camera intrinsics were estimated with Zhang’s planar-pattern calibration [25]. A 6 × 9 black-and-white chessboard (20 mm squares) was printed on A4 paper and fixed flat on the ground. Each camera was then moved manually to capture the pattern from multiple angles and distances. The detected corner points were subsequently refined using nonlinear optimization to recover intrinsic parameters, including focal length, principal point, and distortion coefficients. After calibration, the cameras were installed in fixed positions around the basketball court for subsequent data collection. The system estimated the target’s exact position in the world coordinate system by manually annotating 2D keypoints in the videos and assuming a planar court surface, therefore guaranteeing exact mapping from pixel to world coordinates using the intrinsic and extrinsic parameters of camera.

The Multi-Modal Pose Estimation (MMPose) framework’s RTMPose model, which was used in this study, achieves 75.8% average precision on the COCO val2017 benchmark [21]. Prior research has shown that systems for markerless motion capture based on RTMPose show great agreement with gold-standard marker-based systems [26], showing the algorithm satisfies the technical criteria of this work. Following the completion of 3D scene calibration, the system used the RTMPose toolkit’s detection module (code and models are available at https://github.com/open-mmlab/mmpose (accessed on 11 June 2025)) to identify and crop human bounding boxes in multi-view video frames. This allowed the system to extract body keypoints, including the head, torso, and limb joints and endpoints, in accordance with the COCO Keypoints definition [27]. The Direct Linear Transformation (DLT) algorithm then converted pixel coordinates into world coordinates, producing frame-by-frame 3D skeleton keypoint sequences for every athlete [28].

The markerless motion capture pipeline employed in this study represents a mainstream methodology within the field of computer vision, the accuracy of which has been substantiated by numerous prior investigations [29,30]. Due to the absence of athletes’ ground-truth 3D coordinates, an indirect verification approach was applied: the reconstructed 3D coordinates were reprojected onto original camera views, with the mean reprojection error of approximately 2 pixels (maximum ≤ 5 pixels, camera resolution 1920 × 1080), confirming reliable coordinate estimation.

**Figure 3:**
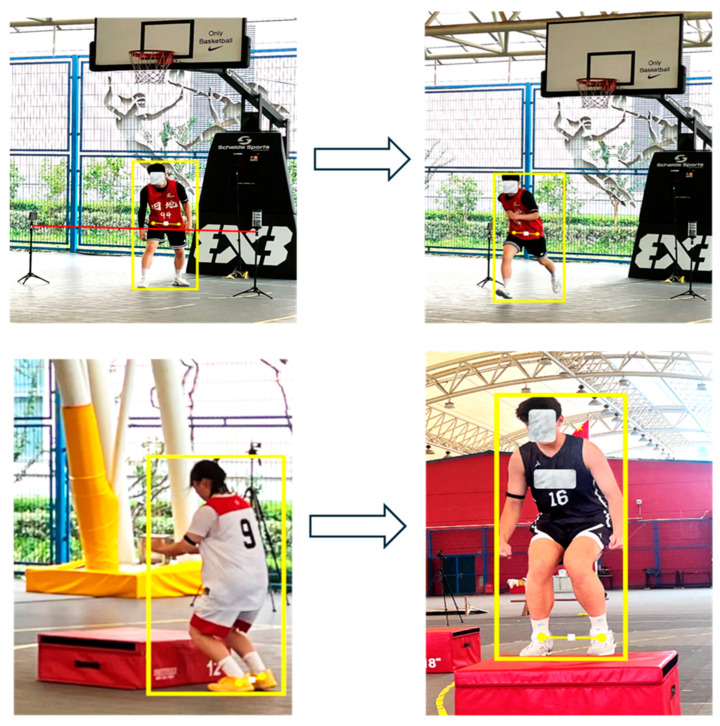
Illustration of keypoint selection for estimating player position in MMC analysis. The hip midpoint was used for horizontal displacement (**top panels**), and ankle midpoint was used for jump height assessment (**bottom panels**).

To simplify subsequent trajectory analysis, the midpoint of the line connecting the left and right hip keypoints was used to represent the player’s position in horizontal displacement calculations. In the analysis of vertical jumping, the athlete’s position was defined as the midpoint between the left and right ankle keypoints (Figure 3). To enhance model robustness and keypoint detection accuracy, we used pre-trained models based on the COCO and AI Challenger datasets; over 2300 3x3 basketball game segments were manually annotated and used for training. The video clips were taken from 98 official games that were recorded during the China Dragon 3x3 Super League in 2024. There were four regional rounds and one final event.

### 2.4. Experiment Procedures

This study drew on previous experimental designs for tracking running distances in team sports and, considering the characteristics of 3x3 basketball and its court structure, designed four sport-specific test tasks: linear running, T-test with multiple movement postures, curved running, and jumping [31,32,33,34,35]. Both the linear and curved running tasks were performed under two conditions: with and without the ball. All predefined distances in the test tasks were measured on-site using a measuring tape, with clear landmarks placed on the court.

**Figure 4:**
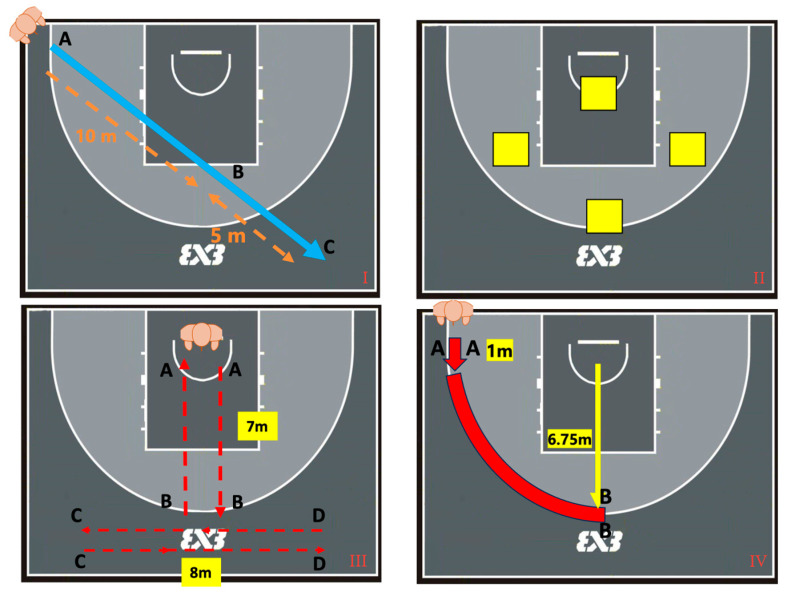
Overview of the experimental tasks and measurement segments for MMC data collection: (**I**) Straight-line runs; A-B acceleration and B-C deceleration. (**II**) Jump tests on plyometric boxes. (**III**) T-test runs; A-B forward, C-D lateral shuffle, and B-A backward. (**IV**) Curved runs.

After a standardized dynamic warm-up, participants completed four basketball-specific drills (Figure 4): (i) straight-line sprint—start at cone A, accelerate 10 m to cone B, and decelerate 5 m to cone C; (ii) jump test—five jumps, each onto 6-, 12-, 18- and 24-inch plyometric boxes; (iii) T-test run—sprint–shuffle–shuffle–sprint–backpedal across markers A–D; (iv) curved run—follow the three-point arc (entry 1 m, radius 6.75 m). Straight and curved runs were executed with and without ball dribbling. Each sprint and jump drill were repeated five times; the T-test and curved runs were repeated three times. All movements were captured synchronously by the eight-camera rig.

### 2.5. Data Collection

Ten participants completed a total of 378 trials. Two of them did not finish the curved running task for personal reasons. We used Python 3.12.2 (packages including pandas, NumPy, SciPy, and Pingouin) for statistical analysis after importing all the data into Excel (v16.96.1).

For horizontal displacement analysis, the study focused on specific segments: 10 m straight-line sprints and 5 m deceleration runs, an 11.60 m curved run comprising a 1 m straight entry and a 6.75 m radius arc, as well as the 7 m straight segment, 8 m lateral shuffle, and 7 m backpedal in the T-test. For vertical movement analysis, jump-height detection accuracy was evaluated by comparing algorithm-identified jumps exceeding each box height with the counts recorded by three human observers (Figure 4).

Time–motion analysis (TMA) was adopted as the criterion measure for segment-specific speed [36]. Two experienced analysts independently marked the video frames at which the athlete’s hip-center crossed each pre-marked court line. To guarantee timestamp consistency, the two analysts cross-checked all annotations and addressed any significant differences by going frame by frame. The elapsed time between successive crossings (Δt, s) was taken as the movement duration for that segment. Segmental speed (v, m·s^−1^) was then calculated using v = d/Δt, where d is the calibrated path length for the drill (10, 5, 7, 8, or 11.6 m). To ensure comparability, the speed measured by the markerless motion capture (MMC) system was also calculated as average speed (v = d/Δt), using the displacement and time data obtained from the MMC system for each movement segment.

### 2.6. Statistical Analysis

For every trial, the coefficient of variation (CV = standard deviation, SD, divided by the mean) of the distance measured by the MMC system was calculated. CV values ≤ 5% were interpreted as “good” practical precision [37,38]. Measurement bias was defined as the absolute error divided by the reference distance, while the standard error of estimate (SEE) represented the standard deviation of this bias.

MMC system-derived running speeds were compared with those obtained from time–motion analysis. The standardized change in mean (SCM), standardized typical error (STE), and intraclass correlation coefficient (ICC) were calculated using Hopkins’ spreadsheet [39]. SCM was interpreted as follows: <0.2 (trivial), 0.2–0.6 (small), 0.6–1.2 (moderate), 1.2–2.0 (large), and >2.0 (very large). An ICC greater than 0.90 was considered excellent agreement [40,41,42].

The Bland–Altman analysis was applied to illustrate the agreement between the MMC and the TMA measurements. Plots display the mean difference (bias) and the 95% limits of agreement (LoA = bias ± 1.96 SD), enabling visual inspection of fixed or proportional bias [43].

## 3. Results

### 3.1. Horizontal-Displacement Validity

Table 1 provides a summary of descriptive accuracy statistics. Across all straight-line tasks, the MMC produced coefficients of variation (CVs) between 0.62% and 2.99%, well below the 5% threshold denoting good practical precision. Mean bias remained ≤1.76% for 10 m and 5 m segments, irrespective of ball possession. The T-test sections yielded CV values of 1.20% (linear sprint), 3.38% (lateral shuffle), and 1.30% (backpedal). Although the lateral shuffle exhibited the greatest bias (4.61%), this error still satisfied the pre-defined acceptability criterion (<5%). Curved-run trials showed the largest dispersion (CV ≈ 4.5%) and bias (≈3.4%), yet retained measurement error within acceptable limits for court-based monitoring.

### 3.2. Average Speed Measurement Consistency

The agreement between MMC and time–motion analysis (TMA) is presented in Table 2. The intraclass correlation coefficients indicated excellent agreement for speed (ICC = 0.97–1.00, 95% confidence interval (CI) 0.86–1.00). The standardized change in mean (SCM) ranged from −0.29 to 0.05, corresponding to trivial (≤0.20) or small (0.20–0.59) effects according to Hopkins’ scale; no moderate or larger effects were detected.

**Figure 5:**
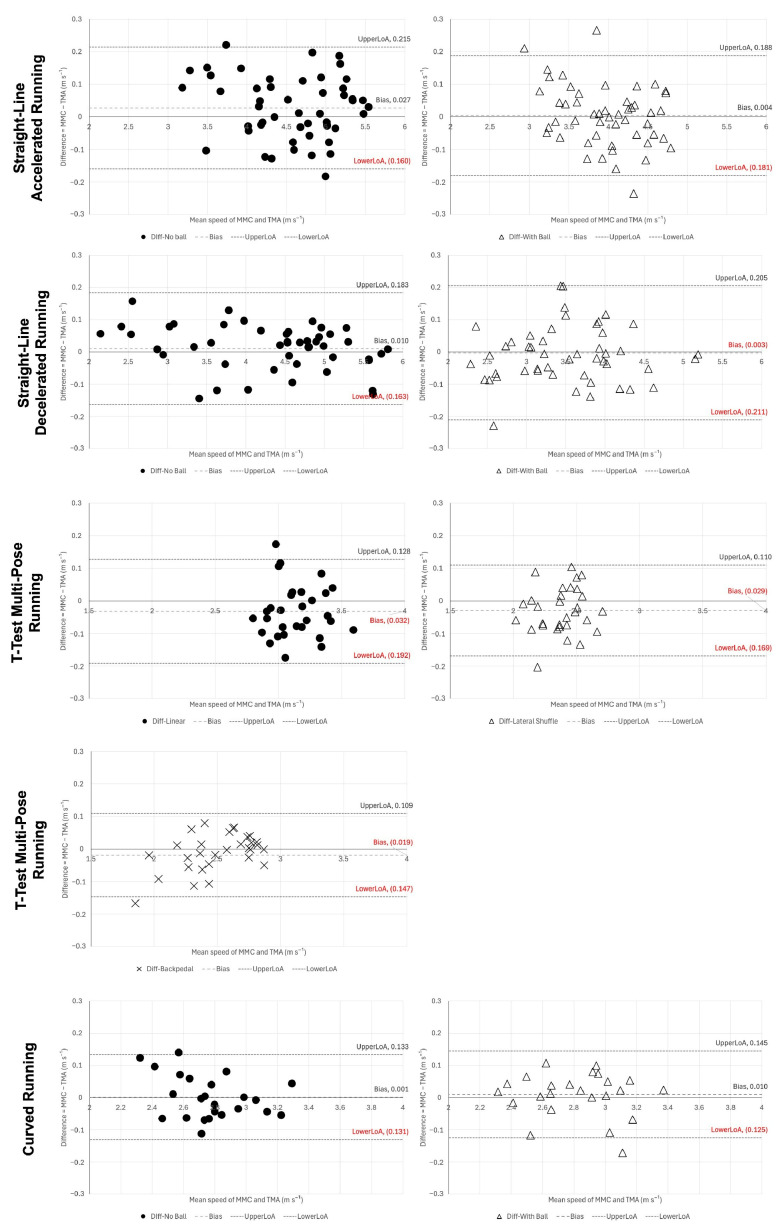
The Bland–Altman plots showing agreement between MMC and TMA for average speed measurements across all movement tasks.

The Bland–Altman plots (Figure 5) demonstrated homoscedastic residuals across the full speed spectrum. Mean bias values were negligible (<0.03 m·s^−1^ for straight-line and curved runs; −0.05 m·s^−1^ for T-test lateral shuffle), with 95% limits of agreement contained within ± 0.42 m·s^−1^. No proportional bias was observed, confirming that MMC speed estimates can be used interchangeably with TMA for all tested movements.

## 4. Discussion

This study is the first to validate a deep-learning MMC workflow directly on an outdoor 3x3 basketball court, thereby moving beyond algorithm-centric, laboratory evaluations, although with only single-player motions. This approach provides a more ecologically valid perspective and extends the practical utility of RTMPose-based systems beyond algorithmic benchmarks.

MMC displayed trivial bias and low coefficients of variation (CVs ≤ 3%) in simple linear movements (acceleration, deceleration, and back-pedal). These results surpass the stability reported for 1 Hz global positioning system (GPS) wearables during 200 m straight-line and curved runs (CV 1.46–6.04%) [17,44,45], confirming that eliminating body-mounted sensors can enhance measurement reliability. The system also has significant practical benefits since it needs no wearable devices and allows for data collection without interfering with the athlete’s natural movement.

The system exhibited better stability during the acceleration segment of the straight-line task, while greater variation was observed during the deceleration segment. This outcome appears to differ from earlier findings indicating that markerless motion capture systems may become less accurate at higher movement speeds [46,47]. One potential explanation is motion blur, which may occur when body segments move faster than the recording system can capture them. This causes inter-frame displacements and lowers keypoint tracking accuracy [48]. In our study, athletes sprinted the same running lane, yet the acceleration segment (10 m) was twice as long as the subsequent deceleration segment (5 m). Although peak speed was reached near the end of acceleration, the deceleration phase required a much higher instantaneous negative acceleration within half the distance. This led to abrupt postural adjustments that further challenged the MMC algorithm’s automatic keypoint identification. These factors may have influenced the observed outcome. Future studies should take this into account when designing experimental protocols.

Under multidirectional tasks, especially lateral shuffles and curved sprints, measurement error rose but stayed within the predetermined 5% acceptable range. The intricacy of these movement patterns could be the cause of this difference. Despite lateral motions like side steps, hops, and lateral runs that are frequently seen in team sports like basketball and volleyball, they are not typical human locomotor patterns. Compared to sagittal-plane movements, lateral displacements involve asymmetrical limb mechanics [49]. As a result, it is difficult for athletes to follow a predefined path consistently. Particularly during lateral shuffles, the actual displacement trajectory often takes the form of a fluctuating curve rather than a straight line. This contributes to the error in displacement and velocity estimation. In the future, we will use gold-standard marker-based systems to compare displacement trajectories during complex movements, to validate the performance of the MMC system.

Displacement error could also come from the assumptions in the experimental design. The hip-center keypoint was consistently used as the tracking reference for horizontal displacement in this study; the moment it crossed a landmark was deemed the sampling point under the assumption the keypoint had no radius and could exactly intersect with the landmark. Especially during the lateral shuffle phase of the T-test, athletes frequently altered direction straight after touching markers C and D with their hand or foot, without the hip-center keypoint crossing the marker. To remedy this, the study designated the sampling point for these two locations as the instant at which the keypoint was closest to the marker. Although the recognition problem was partially resolved by this method, displacement error was introduced, indicating a need for further methodological improvement.

In one of the latest published framework reviews, researchers affirmed the promising potential of 3D markerless motion capture technology in sports. They also pointed out some of its current shortcomings, including occlusion effects, a decrease in accuracy when capturing complex or fast-moving objects, and doubts about how well it adapts to various environmental conditions [50]. Similar to these challenges, though the current results confirm the viability of our approach, three constraints should be noted. First, the setup has not yet been validated under severe player-to-player occlusions or during high-intensity offensive–defensive interactions typical of official 3x3 matches. Second, vertical-plane validation was restricted to a predefined jump-height threshold, preventing precise estimation of continuous vertical displacement and velocity. Third, we did not perform test–retest sessions, which limits conclusions regarding long-term stability and reliability under varying environmental conditions. These problems will be addressed in competitive environments in future research.

## 5. Conclusions

This study designed and field-tested a markerless motion capture workflow for 3x3 basketball that combines multi-camera video with the RTMPose algorithm. When compared with tape-measure and manual time–motion references, the system achieved centimeter-level agreement as well as acceptable repeatability for both horizontal displacements and jump events. Further work is needed to confirm criterion accuracy and ecological validity because the validation drills involved only single athletes in controlled trajectories. Future research should (i) benchmark the workflow against a laboratory-grade optical system, (ii) quantify performance during true game play with multiple athletes, occlusions, and abrupt direction changes, and (iii) investigate portable recording options, such as camera reduction or smartphone arrays to improve courtside practicality.

## Figures and Tables

**Table 1:** Accuracy metrics of horizontal displacement measured by the MMC system across multiple basketball-specific movement tasks. Reference distances were validated using tape measures, and results include CV, bias, and SEE.

Task	N	Condition	Reference Distance (m)	Measured Distance (Mean ± SD, m)	CV (%)	Bias (%)	SEE (%)
**Straight-Line Running**							
10 m Acceleration	50	Without Ball	10	10.045 ± 0.062	0.62%	0.60%	0.48%
10 m Acceleration	50	With Ball	10	10.052 ± 0.137	1.36%	0.96%	0.26%
Average	100	-	10	10.049 ± 0.106	1.06%	0.63%	0.66%
5 m Deceleration	50	Without Ball	5	4.994 ± 0.088	1.76%	1.10%	1.36%
5 m Deceleration	50	With Ball	5	4.957 ± 0.148	2.99%	1.76%	0.31%
Average	100	-	5	4.975 ± 0.123	2.46%	0.92%	1.37%
**T-Test**							
Linear 7 m	30	Without Ball	7	7.017 ± 0.084	1.20%	0.66%	1.03%
Lateral Shuffle 8 m	30	Without Ball	8	8.296 ± 0.28	3.38%	4.61%	2.11%
Backpedal 7 m	30	Without Ball	7	7.068 ± 0.092	1.30%	1.15%	1.16%
Average	-	-	-	-	-	-	-
**Curved Running**							
Curved Running, 11.6 m	24	Without Ball	11.6	11.665 ± 0.523	4.48%	3.42%	2.90%
Curved Running, 11.6 m	24	With Ball	11.6	11.533 ± 0.527	4.57%	3.32%	3.09%
Average	48	-	11.6	11.599 ± 0.518	4.47%	3.37%	2.38%

Note: N, number of trials; measurement results of displacement across different test tasks using the markerless system. Bias (%) = (measured − reference)/reference × 100; SEE (%) = standard deviation of the bias; CV = coefficient of variation. For the T-test, average values are not reported due to varying movement distances across sub-tasks.

**Table 2:** Comparative analysis of average speed measurements between TMA and MMC system across specific basketball tasks. Metrics include SCM, STE, and ICC values.

Task	N	MMC (M ± SD, m/s)	TMA (M ± SD, m/s)	SCM (95%CI)	STE (95%CI)	ICC (95%CI)
Straight-Line Running						
10 m Acceleration (No Ball)	50	4.56 ± 0.47	4.59 ± 0.47	0.05 (0.00~0.10)	0.04 (0.03~0.09)	1.00 (0.99~1.00)
10 m Acceleration (With Ball)	50	3.97 ± 0.31	3.97 ± 0.28	0.05 (−0.06~0.17)	0.10 (0.06~0.20)	0.99 (0.97~1.00)
5 m Deceleration (No Ball)	50	4.33 ± 0.80	4.34 ± 0.79	0.01 (−0.05~0.06)	0.05 (0.03~0.10)	1.00 (0.99~1.00)
5 m Deceleration (With Ball)	50	3.48 ± 0.43	3.48 ± 0.43	−0.02 (−0.14~0.10)	0.10 (0.07~0.21)	0.99 (0.96~1.00)
T-Test						
Linear 7 m	30	3.15 ± 0.16	3.12 ± 0.17	−0.13 (−0.34~0.08)	0.18 (0.12~0.37)	0.98 (0.89~0.99)
Lateral Shuffle 8 m	30	2.39 ± 0.17	2.36 ± 0.16	−0.29 (−0.53~0.04)	0.21 (0.14~0.42)	0.97 (0.86~0.99)
Backpedal 7 m	30	2.51 ± 0.25	2.50 ± 0.28	−0.02 (−0.17~0.12)	0.12 (0.08~0.25)	0.99 (0.95~1.00)
Curved Running						
Curved Running, 11.6 m (No Ball)	24	2.77 ± 0.25	2.77 ± 0.22	0.01 (−0.19~0.20)	0.16 (0.11~0.33)	0.98 (0.92~1.00)
Curved Running, 11.6 m (With Ball)	24	2.81 ± 0.28	2.82 ± 0.28	0.04 (−0.12~0.19)	0.13 (0.09~0.26)	0.99 (0.95~1.00)

Note: N, number of trials; SCM = standardized change in mean; STE = standardized typical error; ICC = intraclass correlation coefficient. SCM thresholds: <0.2 (trivial), 0.2–0.6 (small), 0.6–1.2 (moderate), 1.2–2.0 (large), 2.0–4.0 (very large), and >4.0 (extremely large). ICC > 0.9 indicates high consistency.

## Data Availability

The datasets generated during the current study are available in the Zenodo repository (DOI:10.5281/zenodo.15369748).
